# Posttraining noradrenergic stimulation maintains hippocampal engram reactivation and episodic-like specificity of remote memory

**DOI:** 10.1038/s41386-025-02122-2

**Published:** 2025-05-08

**Authors:** Kubra Gulmez Karaca, Sevgi Bahtiyar, Linde van Dongen, Oliver T. Wolf, Erno J. Hermans, Marloes J. A. G. Henckens, Benno Roozendaal

**Affiliations:** 1 Department of Medical Neuroscience, Radboud university medical center, 6500 HB Nijmegen, The Netherlands; 2 Donders Institute for Brain, Cognition and Behaviour, Radboud University, 6525 EN Nijmegen, The Netherlands; 3 Department of Cognitive Psychology, Ruhr University Bochum, D-44780 Bochum, Germany; 4 Present Address: UMC Brain Center, Department of Translational Neuroscience, University Medical Center Utrecht, Utrecht, Netherlands

**Keywords:** Stress and resilience, Long-term memory, Consolidation

## Abstract

Recent findings indicate that noradrenergic arousal maintains long-term episodic-like specificity of memory. However, the neural mechanism of how norepinephrine can alter the temporal dynamics of systems consolidation to maintain hippocampus dependency of remote memory is currently unknown. Memories are stored within ensembles of neurons that become activated during learning and display strengthened mutual plasticity and connectivity. This strengthened connectivity is believed to guide the coordinated reactivation of these neurons upon subsequent memory recall. Here, we used male transgenic FosTRAP2xtdTomato mice to investigate whether the noradrenergic stimulant yohimbine administered systemically immediately after an episodic-like object-in-context training experience maintained long-term memory specificity which was joined by an enhanced reactivation of training-activated cells within the hippocampus during remote retention testing. We found that saline-treated control mice time-dependently lost their episodic-like specificity of memory, which was associated with a shift in neuronal reactivation from the dorsal hippocampus to the prelimbic cortex at a 14-day retention test. Importantly, yohimbine-treated mice maintained episodic-like specificity of remote memory and retained high neuronal reactivation within the dorsal hippocampus, without a time-dependent increase in prelimbic cortex reactivation. These findings suggest that noradrenergic arousal shortly after training maintains episodic-like specificity of remote memory by strengthening the connectivity between training-activated hippocampal cells during consolidation, and provide a cellular model of how emotional memories remain vivid and detailed.

## Introduction

Extensive evidence indicates that emotionally arousing experiences induce vivid and lasting memories [1, 2]. Memories normally undergo a time-dependent neural reorganization during which their recall becomes gradually less dependent on the hippocampus and more on prefrontal networks [3, 4]. This systems consolidation of memory, which is dependent on hippocampal-prefrontal interactions [5–8], is accompanied by a transformation from originally detailed and specific memory to more semantic gist-like memory [9]. However, arousal-associated noradrenergic activation of the basolateral amygdala (BLA) after an episodic-like training experience was found to maintain hippocampus-dependent specificity of remote memory in rats [10]. Similarly, a functional MRI study in humans demonstrated that post-encoding noradrenergic arousal reduced the decline of memory over time and was associated with increased hippocampal activity as well as reduced hippocampal-prefrontal interplay at remote memory testing [11]. These findings indicate that noradrenergic arousal during the post-learning consolidation phase may actively alter the dynamics of systems consolidation in order to maintain long-term specificity of episodic memory.

Very little is known about the mechanisms mediating this long-term maintenance of hippocampus-dependent specificity of memory by post-learning noradrenergic activation. A recent human neuroimaging study analyzed the pattern of voxel responses in the hippocampus during both memory encoding and retrieval, i.e., encoding-retrieval similarity, for individual items at recent and remote timepoints [11]. Whereas the encoding-retrieval similarity decreased over time under control conditions, noradrenergic arousal post-encoding induced a time-dependent increase in the similarity of the encoding and retrieval responses in the hippocampus, indicating an improved reinstatement of the original memory trace over time [11]. Animal studies have shown that memory traces are encoded by ensembles of neurons that are activated by learning [12–14]. Those neurons, generally referred to as engram cells, undergo learning-induced structural and functional changes that strengthen their preferential synaptic connectivity with each other during memory consolidation [15]. This strengthened synaptic connectivity between engram cells guides their coordinated reactivation during subsequent memory recall [15, 16], which is both necessary and sufficient to evoke memory recall [12, 14] and directly correlates with memory performance [13, 17–20]. This raises the question whether norepinephrine, being a powerful modulator of synaptic plasticity [21–23], may maintain long-term hippocampal dependency of episodic(-like) memory by strengthening the connectivity between engram cells within the hippocampus during the posttraining consolidation period, thereby improving their coordinated reactivation at remote retention testing.

Here, we aimed at investigating whether noradrenergic activation after training on an episodic-like object-in-context (OiC) task maintains high reactivation of engram cells within the dorsal hippocampus at remote retention testing and prevents the time-dependent loss of memory specificity. We used the transgenic FosTRAP2xtdTomato mouse line [24] to label activated cells during the initial training/consolidation period and assessed neuronal responses to a retention test either 3 (recent) or 14 (remote) days later. Previous studies have identified the prelimbic cortex (PL) to be involved in remote memory [5, 25, 26]. For both the dorsal hippocampus and PL, we then computed the reactivation rate (RR), which represents the percentage of cells activated during both the training/consolidation and retention test, expressed as a ratio of the total number of cells activated during the training/consolidation. RRs within the dorsal hippocampus and PL were analyzed for effects of time and noradrenergic stimulation.

## Materials and methods

### Animals

Male heterozygous FosTRAP2xtdTomato mice (8–10 weeks old at the start of behavioral experiments) were used. In this mouse line, systemic administration of 4-hydroxytamoxifen (4-OHT) induces a permanent tdTomato-fluorescent labeling of c-Fos-expressing cells within a 6-h time window [24]. The animals were bred in house by crossing female homozygous Fos2A-iCreER (Fos^tm2.1(icre/ERT2)Luo^/J, #030323, Jackson Laboratory) and male homozygous tdTomato (B6.Cg-Gt*(ROSA)26Sor*^*tm9(CAG-tdTomato)Hze*^/J, #007909, Jackson Laboratory) founder lines. Only male mice were included in this study based on earlier observations of sex differences in object recognition memory per se [27] and sex differences in the noradrenergic system, along with its regulation by emotional arousal [28, 29], which may lead to higher training-induced norepinephrine levels in females [30]. Therefore, proper examination of yohimbine effects on OiC memory in females would likely require specific training or testing at different estrous cycle stages [27, 31–34] and adjustments in experimental conditions, such as different yohimbine dosages or training intensity. Experimental mice were housed individually starting one week prior to commencement of the experiments, under controlled housing conditions (22 °C, light intensity of 47 lux, 72% humidity) and a 12:12-h light:dark cycle (lights on from 07:00–19:00 h). They had *ad libitum* access to water and food. Both training and testing were performed during the light phase of the cycle (between 10:00 and 15:00 h). All procedures were performed in line with European Union Directive 2010/63/EU and were certified by the Central Authority for Scientific Procedures on Animals, The Hague, The Netherlands

### Object-in-context (OiC) task

**Fig. 1:**
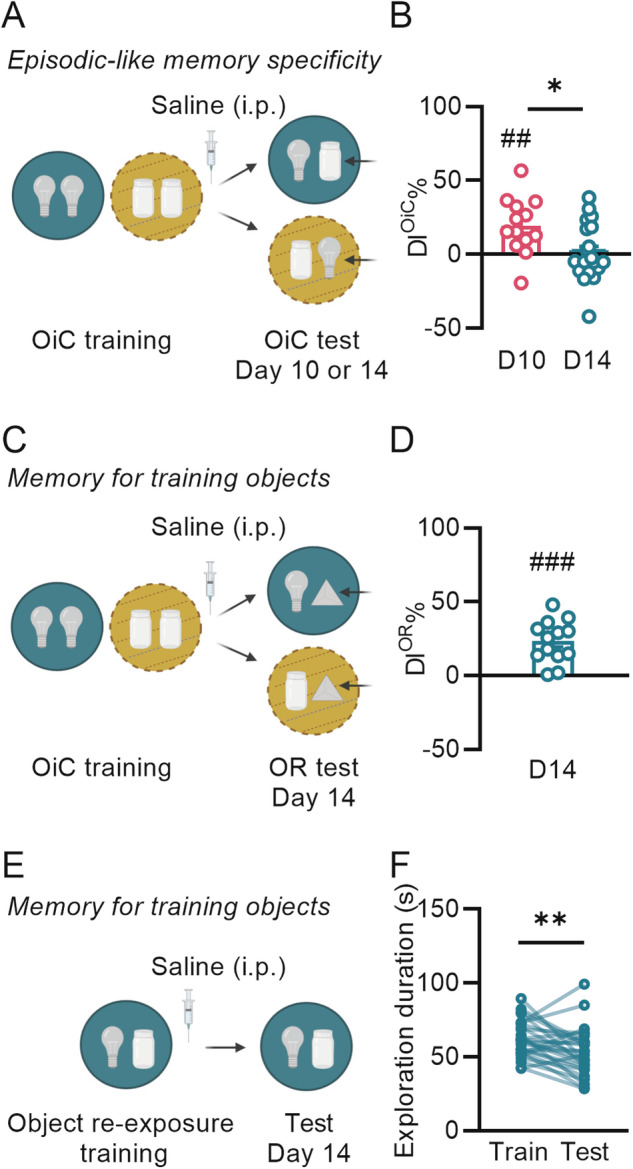
Object-in-context (OiC) memory decays over time in saline-treated mice, while object memory is retained. **A** Schematic representation of the OiC task to assess episodic-like specificity of memory. **B** The discrimination index (DI^OiC^%) of mice at a 10-day (*n* = 13) and 14-day (*n* = 20) retention test (^##^*p* < 0.01 vs chance (0); *p < 0.05). **C** Schematic representation of the object recognition (OR) task to assess memory for the objects per se. **D** The discrimination index (DI^OR^%) of mice at a 14-day retention test (^*###*^*p* < 0.0001 vs chance, *n* = 13). **E** Schematic representatio*n* of the object re-exposure task used to assess memory for the training objects per se. **F** Total object exploration time during training and a 14-day retention test (**p < 0.01, *n* = 27). Data represent mean ± standard error of the mean (s.e.m.) and all data points. Schematic representations are created with BioRender.com.

Episodic-like memory for the association of an object with a specific training context was assessed with an OiC task (Fig. 1A) [35]. Mice were initially handled (5 min per day for 5 days), and then habituated to the training procedures in two contexts, without any objects (3 days, 10 min each), which were different than the two training contexts (see Supplement). On the training session, they were placed in the first context (40 cm in diameter, 40 cm height) where they could explore one set of two identical objects (either two glass jars or two light bulbs) for 10 min. Immediately after the first context exposure, they were placed in a second, distinctly different context (40 cm in diameter, 40 cm height) where they could explore another set of two identical objects for 10 min. Mice trained on a weak OiC protocol were trained for only 5 min in each context. The sequence of the two context exposures and object-context combinations was counterbalanced across animals. Retention of the memory was tested 3 (minimum delay required for detection of tdTomato expression in FosTRAP2xtdTomato mice [24]), 10 or 14 days later by placing the mice in one of the two training contexts for 5 min, which contained one object from each of the two pairs used during the training session. To assess OiC memory, a discrimination index (DI^OiC^%) was calculated as the difference in time spent exploring the novel vs familiar object-context combination, expressed as the ratio of the total time spent exploring both objects (see Supplement). A large DI^OiC^% was interpreted as indicating robust OiC memory.

**Fig. 2:**
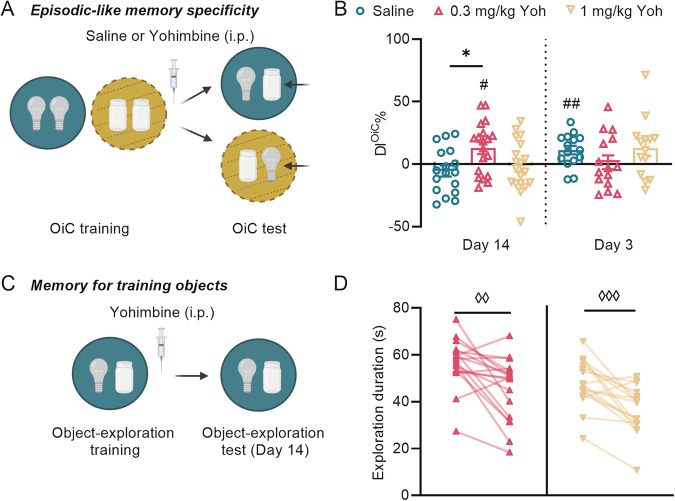
Posttraining noradrenergic stimulation dose-dependently maintains episodic-like memory on an OiC task at a remote memory test, without enhancing recent memory. **A** Schematic representation of the OiC task to examine the effect of posttraining noradrenergic stimulation on episodic-like specificity of memory. **B** The discrimination index (DI^OiC^%) of saline (*n* = 16/19) or yohimbine (Yoh, 0.3 mg/kg (*n* = 15/19) or 1 mg/kg (*n* = 14/19) treated mice at the 3- or 14-day retention test after OiC training (^#^*p* < 0.05, ^##^*p* < 0.01, ^###^*p* < 0.001 vs chance, *p < 0.05, **p < 0.01*)*. **C** Experimental timeline used for assessing the effects of posttraining yohimbine administration on memory for the objects encountered during training. **D** Total exploration time of the objects during training and a 14-day retention test in 0.3 mg/kg (*n* = 18) or 1 mg/kg (*n* = 17) yohimbine-treated mice (^◊◊^*p* < 0.01, ^◊◊◊^*p* < 0.001). Object-context combinations were randomized. Data represent mean ± s.e.m. and all data points. Schematic representations are created with BioRender.com.

Memory for the training objects per se after OiC training, thus independent of the episodic-like association with the training context, was assessed with two different tests: 1) object recognition (OR) test (Fig. 1C) and 2) object re-exposure test (Fig. 1E). Mice were trained on the OiC task as described above. For the OR test (14 days only), they were placed for 5 min in one of the two training contexts (in a randomized manner), which contained one object that was previously encountered in that context and a novel object. Memory for the training object (DI^OR^%) was calculated as the difference in time spent exploring the novel vs familiar object, expressed as the ratio of the total time spent exploring both objects. A large DI^OR^% was interpreted as indicating strong OR memory. For the object re-exposure test (14 days later), mice were re-exposed to one of the two training contexts with the identical object configuration. Total object exploration time during the 10-min testing session was compared with that of the training session (see Supplement). A reduced object exploration was interpreted as mice remembering the objects. Separate groups of mice were used for each task and for each retention interval.

### Systemic drug administration

The noradrenergic stimulant yohimbine (17-hydroxyyohimban-16-carboxylic acid methyl ester hydrochloride; 0.3 or 1.0 mg/kg; Sigma-Aldrich, #Y3125), an α_2_-adrenoceptor antagonist that increases norepinephrine levels in the brain and periphery [36, 37], was dissolved in saline and injected intraperitoneally, in a volume of 0.01 mL/g of body weight, immediately after the training session. Saline was injected as control.

4-OHT (50 mg/kg [24]; Hello Bio Ltd, #HB2508) was injected intraperitoneally to all experimental groups, in a volume of 0.005 mL/g of body weight, immediately following the saline or yohimbine injection. Fifty milligram of 4-OHT was first diluted in 0.5 mL absolute ethanol, sonicated for 1 h at 55–60 °C, and then further diluted in 4.5 mL corn oil and sonicated again for 1 h at 45–55 °C. The final solution contained 10% ethanol and 90% corn oil. All drug solutions were freshly prepared before each experiment.

### Immunohistochemistry

Mice were anesthetized with an overdose of sodium pentobarbital (60 mg/mL, i.p.) 1 h after retention testing, followed by transcardial perfusion with ice-cold phosphate-buffered saline (PBS) and ice-cold 4% paraformaldehyde (PFA, Sigma-Aldrich, pH 7.4). Brains were extracted, post-fixed in 4% PFA overnight at 4 °C, and then transferred to 30% sucrose in PBS for 2–3 days. Coronal sections were cut at a thickness of 20 μm and stored in PBS with 0.01% sodium azide at 4 °C. Four to six sections of both the dorsal hippocampus (anteroposterior (AP), -1.70 to −2.30 mm from Bregma) and PL (AP, +1.98 to +1.54 mm) were selected from each mouse brain. Immunohistochemistry was performed using primary antibody Guinea Pig anti-c-Fos (1:1,000, 226-308, Synaptic Systems) and secondary antibody Donkey anti-Guinea Pig Alexa 647 (1:750, 706-605-148, Jackson ImmunoResearch) (see Supplement).

### Microscopy and image analysis

Images were acquired on a Leica DMI6000B automated high-content microscope with a 20x (dorsal hippocampus) or 10x (PL) magnification. Analyses of the images were performed using FIJI (version 1.53t for Windows [38]). For all images, tdTomato^+^, c-Fos^+^ and tdTomato^+^+c-Fos^+^ cells were counted manually. When analyzing c-Fos immunostaining, a threshold was applied to the processed merged file and particles above the threshold, and larger than 20-pixel units were identified positive after a final confirmation of the cell based on DAPI staining. The threshold was kept constant across all animals within one staining batch, with all batches containing balanced group assignments. For tdTomato^+^ cells, all expressing cells were considered positive, regardless of labeling intensity. See Supplement for further details on the analysis of cells activated by training/consolidation (tdTomato^+^), retention (c-Fos^+^) and/or both (tdTomato^+^+c-Fos^+^).

### Statistical analyses

Statistical analyses were performed using GraphPad Prism for Windows, version 9.4.1. Briefly, if the data followed a normal distribution in Shapiro–Wilk normality test, one-way or two-way ANOVAs were used to compare one or two independent variables, respectively. When appropriate, independent-sample or paired *t*-tests (two-tailed) were used to determine the source of the significance. One-sample *t*-tests were used to determine whether the DI% differed from zero (i.e., chance level) and thus whether the mice displayed memory of the training experience. If the data deviated from a normal distribution, non-parametric alternative tests were used (see Supplement).

## Results

### Episodic-like memory decays over time

As most previous studies investigating the time-dependent loss of episodic-like specificity of memory have employed fear-associative tasks (which per se induce robust endogenous noradrenergic activation), we first determined the temporal dynamics for this decay of much less-arousing OiC memory in saline-treated control mice. We trained FosTRAP2xtdTomato mice on the OiC task (10 min in each context), followed immediately by a systemic administration of saline (Fig. 1A). At a 10-day retention test, mice still displayed episodic-like OiC memory, as indicated by a DI^OiC^% that was significantly greater than zero (one-sample *t*-test: *t*_12_ = 3.68, *p* = 0.003, Fig. 1B). When we extended the testing delay to 14 days in another group of mice, OiC memory was absent (*t*_19_ = 0.28, *p* = 0.79). Moreover, the DI^OiC^% at this 14-day retention test was significantly smaller than that on the 10-day retention test (independent samples *t*-test*: t*_31_ = 2.67, *p* = 0.01, Fig. 1B). Memory for the training objects per se, thus independent of the episodic-like association with the training context, after OiC training was still present at the 14-day retention test (OR test: one-sample *t*-test: *t*_12_ = 5.91, *p* < 0.0001 (Fig. 1C, D); object re-exposure test: paired *t*-test*: t*_26_ = 2.95, *p* = 0.007 (Fig. 1E, F)). These findings thus indicate that selectively the episodic-like aspect of OiC memory was compromised in saline-treated mice at a 14-day retention test, whereas they still displayed memory of the training objects themselves.

### Posttraining noradrenergic stimulation maintains episodic-like memory

Next, we examined whether posttraining noradrenergic activation maintains episodic-like memory at this 14-day retention test. For this, mice were trained on the OiC task, followed immediately by the administration of yohimbine (0.3 or 1 mg/kg) or saline (Fig. 2A). Retention was tested 14 days later. A one-way ANOVA for the DI^OIC^% indicated a significant yohimbine effect (*F*_2,52_ = 4.33, *p* = 0.02). The DI^OiC^% of the 0.3 mg/kg yohimbine group was significantly larger than that of the saline group (*post-hoc* Tukey test: *p* = 0.02), whereas the DI^OiC^% of the 1 mg/kg yohimbine group did not significantly differ from that of saline-treated controls (*p* = 0.09, Fig. 2B). Further, the 0.3 mg/kg yohimbine group had a DI^OiC^% that was significantly greater than zero (one-sample *t*-test: *t*_18_ = 2.71, *p* = 0.01), indicating successful memory recall, whereas the DI^OiC^% of mice treated with either saline or 1 mg/kg yohimbine did not differ from zero (saline: *t*_16_ = 1.24, *p* = 0.23; 1 mg/kg: *t*_18_ = 0.29, *p* = 0.77). Consistent with the analysis of DI^OiC^%, the 0.3 mg/kg yohimbine group spent significantly more time exploring the object novel to the testing context (Supplementary Fig. S2B), whereas mice treated with saline or 1 mg/kg yohimbine spent similar time exploring the two objects (Supplementary Fig. S2B). The total time spent exploring both objects during the retention test (Supplementary Fig. S2C) or the total distance traveled in the testing context (Supplementary Fig. S3A) was similar across groups. All groups showed significant memory for the training objects per se, as indicated by a significant reduction in object exploration during an object re-exposure test (RM two-way ANOVA: re-exposure effect: *F*_*1*,59_ = 37.74, *p* < 0.0001) without a yohimbine (*F*_2,59_ = 2.08, *p* = 0.13) or yohimbine*re-exposure interaction effect (*F*_2,59_ = 0.68, *p* = 0.51) (Fig. 2C, D). These findings indicate that posttraining noradrenergic activation dose-dependently maintains episodic-like specificity of memory on an OiC task that normally decays at a remote timepoint.

### Noradrenergic activation differentially impacts recent and remote OiC memory

We then examined whether this effect of posttraining noradrenergic activation is the result of an initial strengthening of the memory. To this end, separate groups of mice treated with yohimbine (0.3 or 1 mg/kg) or saline after OiC training (10 min in each context) were tested for retention 3 days after the training session (Fig. 2A). Yohimbine treatment did not increase the DI^OiC^% at this 3-day retention test (*F*_2,41_ = 1.38, *p* = 0.26, Fig. 2B). In fact, yohimbine seemed even to impair memory as the DI^OiC^% of both yohimbine treatment groups did not differ significantly from zero (0.3 mg/kg: *t*_14_ = 0.27, *p* = 0.79; 1 mg/kg: *t*_13_ = 1.99, *p* = 0.07), whereas the DI^OiC^% of saline-treated mice was significantly greater than zero (*t*_14_ = 3.30, *p* = 0.005, Fig. 2B). Furthermore, saline-treated mice showed preferential exploration of the novel relative to the familiar object in the testing context, whereas yohimbine-treated groups spent similar time exploring the two objects, consistent with the DI^OiC^% analysis (Supplementary Fig. S2B). These findings thus indicate that the effect of posttraining noradrenergic stimulation on the long-term maintenance of episodic-like memory cannot be explained by an initial strengthening of that memory.

Since posttraining noradrenergic stimulation was previously shown to enhance recent OiC memory [39], we next wanted to confirm that a weaker OiC training protocol would lead to memory enhancement upon noradrenergic stimulation. Therefore, we trained mice on the OiC task for only 5 min, creating weak memory in control mice, followed by immediate posttraining administration of yohimbine (0.3 or 1 mg/kg) or saline (Supplementary Fig. S2D). As expected, whereas DI^OiC^% of saline-treated mice did not differ significantly from zero on a 3-day retention test (*t*_16_ = 1.70, *p* = 0.11), the DI^OiC^% of the 0.3 mg/kg yohimbine group was significantly greater than zero (0.3 mg/kg: *W* = 190, *p* < 0.0001; 1 mg/kg: *t*_19_ = 1.37, *p* = 0.19), indicating robust OiC memory. In line with the DI^OiC^% analysis, mice administered 0.3 mg/kg yohimbine spent more time exploring the novel object in that context, while saline- or 1 mg/kg yohimbine-treated groups spent similar time exploring the two objects (Supplementary Fig. S2B). The total time spent exploring both objects during the 3-day retention tests (Supplementary Fig. S2C) or the total distance traveled in the testing context (Supplementary Fig. S3) was similar across groups. These findings thus confirm that posttraining noradrenergic stimulation enhances the consolidation of weaker memories.

### Time-dependent changes in neuronal reactivation within hippocampal-PL networks

**Fig. 3:**
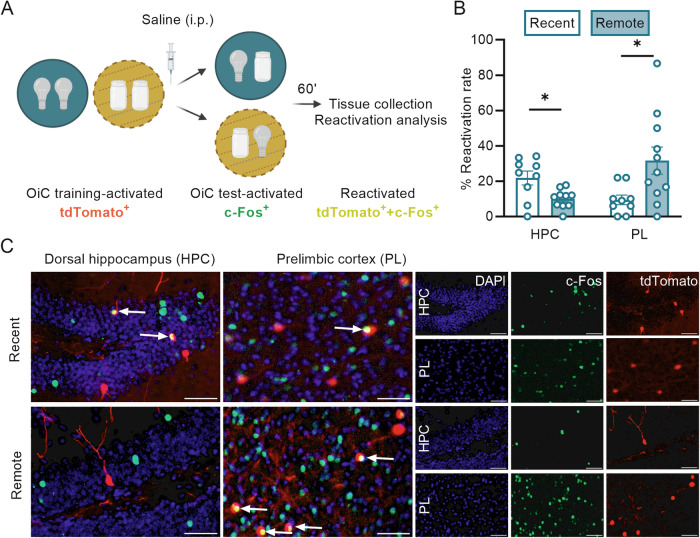
OiC memory in saline-treated mice endures a time-dependent shift in neuronal reactivation from the dorsal hippocampus to the prelimbic cortex networks. **A** Schematic representation of the experimental procedure used to assess the reactivation rate during the OiC retention test. **B** Analysis of the reactivation rate in the dorsal hippocampus (HPC, *n* = 9/10) and PL (*n* = 9/11) during the recent vs remote retention test (*p < 0.05). **C** Representative images of tdTomato^+^ (training^-^activated), c-Fos^+^ (retention^-^activated), and tdTomato^+^+c-Fos^+^ (reactivated) cells in the dorsal hippocampus (left image and upper line of single-channel images) and PL (right image and lower line of single-channel images) of saline-treated mice at recent and remote time point analysis. Scale bar represents 50 µm. Arrows point to examples of reactivated cells. Schematic representations are created with BioRender.com.

We next investigated whether the time-dependent loss of episodic-like OiC memory in saline-treated control animals was associated with a shift in neuronal reactivation from the dorsal hippocampus to the PL. We first confirmed that the labeling system of FosTRAP2xtdTomato mice was strictly dependent on 4-OHT administration (Supplementary Fig. S4), and sensitive enough to capture increased neuronal activation in the dorsal hippocampus and PL following OiC training (Supplementary Fig. S4). We then examined neuronal activity in the dorsal hippocampus and PL during both OiC training/consolidation (tdTomato^+^) and retention test (c-Fos^+^), as well as the RR of training-activated cells (%tdTomato^+^+c-Fos^+^ of the total tdTomato^+^ population) at a recent (3-day) vs remote (14-day) retention test (Fig. 3A–C).

A mixed-effects RM ANOVA for the RR revealed a significant interaction effect between brain region and testing delay (*F*_1,14_ = 13.68, *p* = 0.002), indicating that the RR in the dorsal hippocampus and PL was differentially affected over time. Further analyses revealed a significant time-dependent reduction in the RR within the dorsal hippocampus (independent samples *t*-test: *t*_17_ = 2.81, *p* = 0.01, Fig. 3B), and a significant increase within the PL (*t*_18_ = 2.50, *p* = 0.02, Fig. 3B), indicating a time-dependent shift in the RR from the dorsal hippocampus to PL in saline-treated mice. Noteworthy, the RR in both brain regions at both retention tests was still higher than expected based on chance (Supplementary Fig. S5A, B), suggesting that both brain regions were still engaged at the 14-day retention test. The total number of c-Fos^+^ cells in the dorsal hippocampus or PL that was activated during either the 3-day or 14-day retention test was not different (Supplementary Fig. S5C, D), indicating that this time-dependent reorganization was specific to the reactivation of those cells that were also active during training. Correlational analyses further revealed that the RR in both brain regions was not dependent on the total number of cells that was activated during either training or retention testing (Supplementary Fig. S5E-H). These findings thus indicate that OiC memory endures a time-dependent shift in the RR, in which the reactivation decreases within the dorsal hippocampus over time, whereas it increases within the PL.

### Noradrenergic stimulation evokes sustained hippocampal reactivation

We then assessed whether posttraining yohimbine administration prevents this time-dependent shift in the RR from the dorsal hippocampus to the PL. In these analyses, we only examined the 0.3 mg/kg dose of yohimbine, i.e., the dosage that maintained long-term episodic-like OiC memory. A mixed-effects RM ANOVA for the RR of yohimbine- and saline-treated mice demonstrated a significant interaction between brain region and testing delay (*F*_1,34_ = 17.41, *p* = 0.0002), and a trend-level significant yohimbine*brain region*testing delay interaction effect (*F*_1,34_ = 3.41, *p* = 0.07). There was no yohimbine*brain region interaction effect (*F*_1,34_ = 0.53, *p* = 0.47). As a secondary analysis, we separately investigated the effects of yohimbine on time-dependent changes of the RR within the hippocampus and PL.

**Fig. 4:**
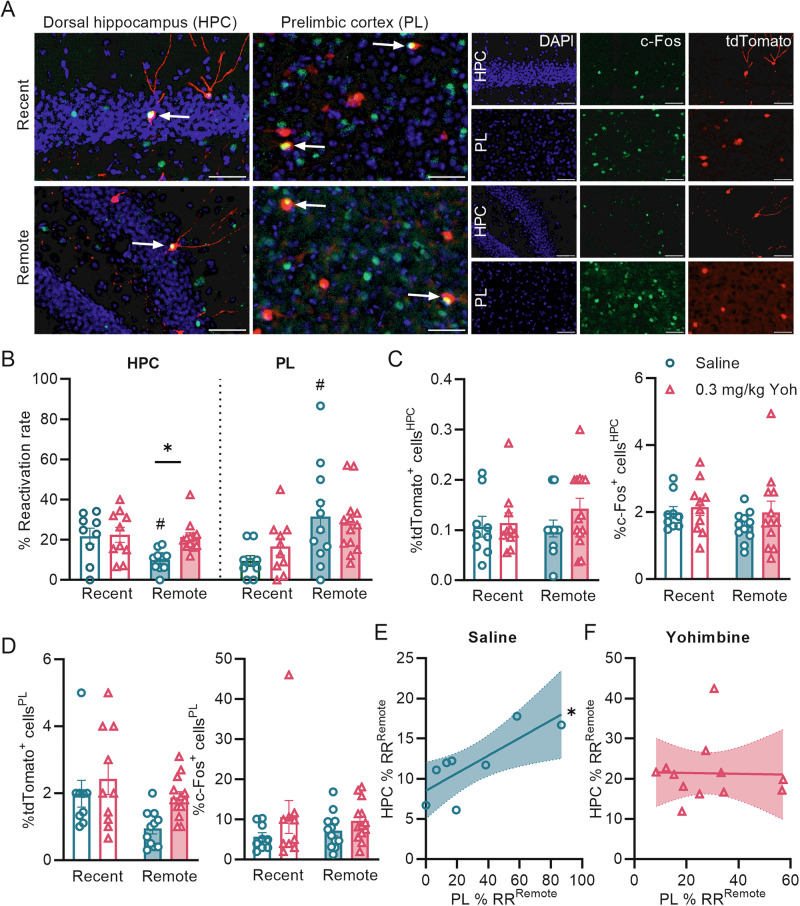
Noradrenergic stimulation evokes sustained reactivation within the dorsal hippocampus and reduces hippocampal-prelimbic cortex correlated reactivation at remote OiC memory recall. **A** Representative images of tdTomato^+^ (training-activated), c-Fos^+^ (retention-activated), and tdTomato^+^+c-Fos^+^ (reactivated) cells in the dorsal hippocampus (left image and upper line of single-channel images) and PL (right image and lower line of single-channel images) of 0.3 mg/kg Yohimbine-treated mice at recent and remote time point analysis. Scale bar represents 50 µm. Arrows point to examples of reactivated cells. **B** Reactivation rate within the dorsal hippocampus and PL in saline- vs yohimbine-treated mice (dorsal hippocampus *n* = 10/12, PL *n* = 10/13) over time (yohimbine: *p < 0.05*;* time: ^*#*^*p* < 0.05). **C** Percentage of tdTomato^+^ or c-Fos^+^ cells in the dorsal hippocampus of saline- vs yohimbine-treated mice at the recent (3-day) or remote (14-day) retention test. **D** Percentage of tdTomato^+^ or c-Fos^+^ cells in the PL of saline- vs yohimbine-treated mice at 3-day or 14-day retention testing. **E** Correlational analysis between the reactivation rates (RR) in the dorsal hippocampus vs PL of saline-treated mice at the 14-day retention test (*p < 0.05*, n* = 8). **F** Correlational analysis of the reactivation rates in the dorsal hippocampus vs PL of 0.3 mg/kg yohimbine-treated mice at the 14-day retention test (*p < 0.05, *n* = 12). Data represent mean ± s.e.m. and all data points.

For the dorsal hippocampus, a two-way ANOVA for the RR revealed significant main effects of yohimbine (*F*_1,37_ = 4.12, *p* = 0.049) and testing delay (*F*_1,37_ = 4.73, *p* = 0.04), without significant yohimbine*testing delay interaction (*F*_1,37_ = 3.17, *p* = 0.08, Fig. 4A, B). While yohimbine-treated mice showed a similar RR within the dorsal hippocampus over time (*post-hoc* Sidak test: *p* = 0.95 3-day vs 14-day retention test), saline-treated mice showed a time-dependent reduction in hippocampal RR (*p* = 0.02, Fig. 4B). The RR of yohimbine- and saline-treated mice did not differ at the 3-day retention test (*p* = 0.98), while yohimbine-treated mice displayed a significantly greater RR at the 14-day retention test (*p* = 0.02). Yohimbine did not affect the total number of tdTomato^+^ (Yohimbine: *F*_1,40_ = 1.40, *p* = 0.24; testing delay: *F*_1,40_ = 0.39, *p* = 0.53; yohimbine*testing delay*: F*_1,40_ = 0.65, *p* = 0.42, two-way ANOVA) or c-Fos^+^ cells (Yohimbine: *F*_1,40_ = 1.43, *p* = 0.24; testing delay: *F*_1,40_ = 1.22, *p* = 0.27; yohimbine*testing delay*: F*_1,40_ = 0.28, *p* = 0.59) in the dorsal hippocampus at either testing delay (Fig. 4C).

For the PL, a two-way ANOVA for the RR indicated no main effect of yohimbine (*F*_1,39_ = 0.13, *p* = 0.72) or yohimbine*testing delay interaction (*F*_1,39_ = 0.99, *p* = 0.32, Fig. 4B), but a significant main effect of testing delay (*F*_1,39_ = 10.33, *p* = 0.003). *Post-hoc* Sidak test revealed that the RR of yohimbine-treated mice remained similar at both timepoints (*p* = 0.21, Fig. 4B), whereas that of saline-treated mice was increased at the 14-day retention test (*p* = 0.01). A two-way ANOVA for the total number of tdTomato^+^ cells revealed a main effect of yohimbine (two-way ANOVA: *F*_1,39_ = 5.02, *p* = 0.03) and testing delay (*F*_1,39_ = 6.72, *p* = 0.01), and no yohimbine*testing delay interaction (*F*_1,39_ = 0.62, *p* = 0.44); however, *post hoc* analyses could not confirm statistical significance at either timepoint (Fig. 4D). The total number of c-Fos^+^ cells within the PL was not affected by yohimbine at either of the retention test delays (Yohimbine: *F*_1,40_ = 2.80, *p* = 0.10; testing delay: *F*_1,40_ = 0.02, *p* = 0.88; yohimbine*testing delay*: F*_1,40_ = 0.32, *p* = 0.57, Fig. 4D).

### Noradrenergic stimulation reduces hippocampal-PL coordinated reactivation

The systems consolidation of memory and time-dependent maturation of engram cells in the PL requires a functional interaction between the hippocampus and PL, particularly at the later stages of systems consolidation [5, 6, 25]. Human studies indicated that yohimbine administration reduced this hippocampal-inferior frontal gyrus functional connectivity [11]. Therefore, we calculated correlations between the RR in the dorsal hippocampus and PL at both 3-day and 14-day retention tests. Consistent with previous findings demonstrating that this hippocampal-prefrontal functional connectivity gradually develops over time [11], we found a positive correlation between the RRs in the dorsal hippocampus and PL of saline-treated mice at the 14-day retention test (Pearson correlation test: *r*_8_ = 0.78, *p* = 0.02, Fig. 4E, Supplementary Fig. S6A), but not at the 3-day retention test (*r*_8_ = 0.48, *p* = 0.24). In contrast, yohimbine-treated mice did not display a significant correlation at either the 3-day (*r*_10_ = 0.41, *p* = 0.24) or 14-day retention test (*r*_12_ = −0.02, *p* = 0.95, Fig. 4F, Supplementary Fig. S6B). Furthermore, the strength of this correlation of yohimbine-treated mice was significantly weaker than that of saline-treated mice at the 14-day retention test (*Z* = 1.91, *p* = 0.03), whereas it did not differ between both groups at the 3-day retention test (*Z* = 0.15, *p* = 0.44). Thus, posttraining noradrenergic activation reduced hippocampal-PL coordinated reactivation at remote memory recall.

## Discussion

Our findings indicate that yohimbine administration after OiC training maintains long-term hippocampus-dependent specificity of remote memory. Further, yohimbine prevented the time-dependent shift in reactivation of training-activated cells from the dorsal hippocampus to the PL observed in saline-treated mice, and maintained a high neuronal reactivation within the dorsal hippocampus without altering overall dorsal hippocampal activity upon retention. These findings support the view that noradrenergic activation maintains episodic-like specificity of remote memory by actively altering the dynamics of systems consolidation, and may represent a critical mechanism underlying the long-term vividness of emotional memories.

Yohimbine increases norepinephrine release in the brain, inducing a state of arousal and anxiety [40]. Thus, the use of posttraining yohimbine administration immediately after the training to mimic a heightened emotional arousal state during the initial consolidation period, excludes potential influences on, for example, attentional, sensory or motivational processes during the training session that may directly affect task performance or promote the prioritization of the most salient features in memory. Here, we found that noradrenergic activation during the initial consolidation window dose-dependently enhanced the consolidation of a weak, but not strong, episodic-like training that normally resulted in no-long term memory during 3-day retention testing mice. These findings confirm a large body of literature that the effects of neuromodulatory agents on memory follow an inverted U-shaped dose-response relationship and depend on the baseline performance of subjects [41–50]. Our finding that noradrenergic activation during the initial consolidation process altered the dynamics of systems consolidation and, as a consequence, maintained long-term episodic-like specificity of memory perfectly aligns with findings of previous studies. Norepinephrine administration into the BLA immediately after training on an inhibitory avoidance discrimination task, comprising the subsequent exposure to two distinct training contexts, of which one was associated with footshock delivery, was found to maintain long-term hippocampus-dependent episodic-like specificity of memory [10]. At a 28-day retention test, saline-treated rats showed loss of episodic-like memory for the association of shock with the specific training context. In contrast, norepinephrine-treated rats continued to display specific memory of the shock-context association. Inactivation of the hippocampus at this remote retention test blocked the display of episodic-like specificity of memory [10], indicating the norepinephrine treatment during the initial consolidation process had maintained long-term hippocampal involvement in the memory. Consistent with these findings, a recent neuroimaging study in humans showed that noradrenergic stimulation post-encoding resulted in a time-dependent increase in hippocampal activity and decrease in neocortical activity during remote retention testing [11].

By using an episodic-like OiC task [35], we here demonstrate that posttraining noradrenergic stimulation maintains a high reactivation of encoding-activated cells in the dorsal hippocampus at remote memory recall. Previous findings have indicated that such reactivation of encoding-activated cells, often referred to as engram cells, relies on a strengthening of their mutual synaptic connectivity during memory consolidation [12, 15, 16]. Reactivation of these cells during the retention test was further proven to be necessary to trigger memory recall [13, 14]. Here, we found that posttraining noradrenergic activation did not influence overall dorsal hippocampal activity at either training/consolidation and/or the retention test. Norepinephrine, via the activation of β-adrenoceptors, is a powerful neuromodulator of synaptic plasticity [15, 27, 34], and shown to increase the phosphorylation of glutamate receptors [51] and facilitate AMPA receptor trafficking in activated synapses [52]. Interestingly, norepinephrine has been suggested to specifically favor the strengthening of activated synapses, amplifying “high-priority” neural representations while simultaneously blocking low-priority signals [53]. Moreover, norepinephrine in the presence of training-induced glutamatergic activity has been proposed to induce the release of additional norepinephrine from adjacent pre-synaptic boutons, which prolonged the window of excitation of target neurons by temporarily inhibiting the afterhyperpolarization [53]. A selective role of norepinephrine in strengthening neural plasticity of activated synapses would thus be consistent with our findings that posttraining yohimbine administration selectively enhanced the reactivation of training-activated cells, without influencing total dorsal hippocampal activity at either training/consolidation or retention test. This could be a possible mechanism of how posttraining noradrenergic activity maintains the high reactivation of encoding-activated cells, presumable engram cells, within the dorsal hippocampus.

Along with a strengthening of synaptic connectivity between presumed engram cells during the initial consolidation period, norepinephrine might also maintain long-term plasticity of these engram synapses by increasing their metaplasticity [23]. Metaplasticity implies that synaptic plasticity changes upon neuronal activity alters the excitability state of these synapses in a persistent manner. This primes them for future long-term plasticity processes [54] that are distinct from the synaptic plasticity processes that underlie the initial strengthening of memory. In support of this view, a previous study had demonstrated that norepinephrine administration into the BLA after an episodic-like training experience actively maintained high levels of Reelin expression, a plasticity-inducing protein [55], in the hippocampus even 28 days later by sustaining reduced methylation of the promotor region of *Reln*, as well as decreased levels of de novo DNA methyltransferases [10]. Interestingly, norepinephrine-treated rats in that study initially displayed lower Reelin expression in the hippocampus compared to saline controls, but progressively increased their levels over time, supporting a metaplastic effect. In accordance with this, Reelin signaling was proposed to promote further stability and maintenance of synapses, hence metaplasticity, in the adult hippocampus [56, 57]. Such an enduring effect of norepinephrine on synaptic plasticity is also consistent with the findings of the earlier described human neuroimaging study indicating that yohimbine treatment induced a progressive increase in encoding-retrieval pattern similarity in the hippocampus, in which encoding-related hippocampal pattern representations (i.e., voxel responses) were reinstated during remote, but not recent, memory recall [11, 58]. Whereas human neuroimaging lacks the spatial resolution to assess the activity of individual cells, or ensembles of cells [59], the present study provides experimental evidence that selectively the encoding-activated neuronal ensembles are reinstated during remote memory recall. This work, therefore, collectively perfectly exemplifies how norepinephrine can progressively facilitate synaptic plasticity within the hippocampus, both at the molecular and population level, to actively maintain, or even strengthen, long-term connectivity of encoding-activated cells. As such, this mechanism could modulate remote memory by rendering engram cells within the hippocampus resistant to systems consolidation. This long-term effect appears to be independent from an initial strengthening the memory, supported by our behavioral finding that yohimbine-induced long-term maintenance of OiC memory was not associated with an initial strengthening of the memory.

Whereas memory for the object-context association is dependent on the hippocampus, recognition of the objects per se relies on different cortical structures, including the PL [60–62]. During systems consolidation, functionally active engram cells within the hippocampus project to functionally immature engram cells within the PL which do not yet contribute to memory recall [5, 25], allowing for their time-dependent maturation and involvement in remote memory recall [5, 25, 26]. This maturation and engagement of PL neurons during remote memory recall is critical for the development of abstract, generalized knowledge concepts [9], and the gradual formation of semantic memory associations in humans [55]. Consistent with such a hippocampal-PL interplay in the systems consolidation process, we found that saline-treated control animals displayed a time-dependent increase in the RR in the PL and that the correlated reactivation (proposedly representing their communication) of dorsal hippocampal-PL circuits increased over time. Importantly, yohimbine administration blocked this correlated reactivation of dorsal hippocampal-PL circuits, which aligns with the previous observation in humans that posttraining noradrenergic stimulation reduced the time-dependent increase in hippocampal-prefrontal functional connectivity, as well as prevented the semantic transformation of episodic memories to prefrontal (and parietal) representations [11, 58]. Furthermore, other studies have indicated that sharp wave-ripple complexes within the hippocampus heavily modulate neocortical events, including the ones in the PL [63, 64] and thus regulate the coupled, synchronized hippocampal-PL activity [63–65]. Noradrenergic activation of the hippocampus was found to suppress hippocampal sharp wave-ripple complexes [66] and to decrease such synchronized activity of the hippocampus and neocortex [65]. These findings therefore suggest that norepinephrine-induced synaptic plasticity within the hippocampus may have consequences at the systems level and impact functional interactions with the PL over time, decelerating the systems consolidation of memory.

In conclusion, this study revealed that noradrenergic activation not only prolongs the episodic-like specificity of an object-associative memory, but also modulates the recruitment of encoding-activated cells in prefrontal-hippocampal circuits over time. These findings suggest that noradrenergic activation can impact mnemonic specificity by enhancing the synaptic plasticity and connectivity of presumed engram cells within the hippocampus. It should be noted that not all stress modulators affect long-term episodic-like specificity of memory in a similar fashion. Previous findings have shown that the stress hormone corticosterone induced the opposite effect and facilitated the systems consolidation process and promoted the generalization of memory [67–71]. Future studies should explore how these different stress modulators interact to determine specificity vs generalization of memory and regulate the long-term fate of emotional memories.

## Supplementary information

Supplementary Data for Posttraining noradrenergic stimulation maintains hippocampal engram reactivation and episodic-like specificity of remote memory

## Data Availability

All data will be made available upon request.
