# Supplementary material for: Posttraining noradrenergic stimulation maintains hippocampal engram reactivation and episodic-like specificity of remote memory
Source: Neuropsychopharmacology. 2025 May 8;50(12):1845–54. doi: 10.1038/s41386-025-02122-2 (PMC12518655; doi:10.1038/s41386-025-02122-2)
Supplement: Supplementary file 1 — Supplementary Data for Posttraining noradrenergic stimulation maintains hippocampal engram reactivation and episodic-like specificity of remote memory [file 41386_2025_2122_MOESM1_ESM.docx]

**Supplementary Data for**

**Posttraining noradrenergic stimulation maintains hippocampal engram reactivation and episodic-like specificity of remote memory**

**This file includes:**

**Supplementary Materials and Methods**

**Supplementary Figures S1-S6**

**Supplementary Materials and Methods**

***Object-in-context (OiC) task***

Episodic-like memory for the association of an object with a specific training context was assessed with an OiC task. Mice were first habituated to the experimenter and experimental room by gentle handling for 5 min per day on 5 consecutive days. Subsequently, the animals received 3 days of habituation to reduce novelty stress which is required to guarantee sufficient exploration of the objects on the training session. Mice were habituated to two boxes for 10 min each. Habituation boxes were different in shape than training boxes to prevent prior encoding of the training contexts in order to ensure maximal involvement of the hippocampus during training. Specifically, habituation boxes were square (40 x 40 x 40 cm), whereas training boxes were round (40 cm in diameter, 40 cm in height). One of the habituation boxes had corncob bedding and contextual modifications on the walls (i.e., a dotted pattern), while the second box contained sawdust bedding and had no contextual modifications. Therefore, the two habituation boxes differed in tactile and contextual aspects. The walls and bedding materials of the round training boxes were matching the habituation boxes, i.e., one having corncob bedding and contextual modifications, while the other having sawdust bedding and no modifications. During the habituation, the animals could explore each context without any objects. The next day, mice were trained on the OiC task to acquire an association between a particular set of objects and a certain context. On the training session, the mice were placed in the first box, and were able to explore one set of two identical objects for 10 min (or 5 min during weak OiC training). Immediately after the first context exposure, mice were placed in the second box for 10 min, containing another set of two identical objects. The objects were two white glass light bulbs (6 cm diameter, 11 cm length) and two transparent glass vials (5.5 cm diameter, 5 cm height), secured to the floor of the boxes with Velcro tape. Previous findings of our lab indicated that a 10-min training session induces robust recent memory in control animals. For retention testing, either 3, 10 or 14 days later, mice were placed in one the two training contexts for 5 min, which now contained one object from each of the two pairs used during the training session. Combinations of objects and contexts, locations of the objects, the order of context exposures during training, and the context used on the retention test were fully randomized across animals. Days 10 and 14 were chosen for retention testing because OiC memory, based on neutral object-context associations, is expected to be shorter lasting compared to the memory generated in fear conditioning paradigms commonly employed in the literature (and suitable for testing at 3-4 weeks post-training). This timing allowed us to pinpoint when OiC memory was lost while object memory remained intact. Total object exploration time during the training session was not different for the two training objects or the two training contexts and also not affected by context order (Supplementary Figure 1A-C). Further, mice spent similar time exploring the two training objects, the two contexts, and first vs. second context. Total object exploration time during training was also similar for the different posttraining drug treatment groups (Supplementary Figure 1E-H). Training and test sessions were videotaped and object exploration times analyzed by a researcher blind to the experimental condition. Object exploration was defined as actual active interaction with an object, i.e., pointing the nose to the object at a distance of <1 cm and/or touching it with the nose. Turning around, climbing or sitting on an object *per se* was not included in exploration time. To avoid the skewing of results that may originate from insufficient or biased exploration, we implemented a standard exclusion criteria for animals displaying extremely low exploration times during the retention test (< 2 s) as this could generate very noisy, and potentially unreliable results, or animals displaying a strong preference (> 67%) to one of the objects during the training session. Of note, no mice were excluded due to insufficient exploration during the retention test, whereas two mice from the weak OiC training were excluded due to a preference to one of the objects during training. To analyze OiC memory, a discrimination index (DI^OiC^%) was calculated as the difference in time exploring the novel and familiar object-in-context combination, expressed as the ratio of the total time spent exploring both objects (i.e., (Time Novel - Time Familiar) / (Time Novel + Time Familiar) x 100%). A large DI^OiC^% was interpreted as robust OiC memory.

***Object recognition (OR) test***

Memory for the training objects *per se* after OiC training, thus independent of the episodic-like association with the training context, was assessed with an OR test. Mice were handled (5 min per day for 5 days), habituated (3 days, 10 min each) and trained (10 min in each context) the same way as in the OiC task. For the OR test (14 days only), mice were placed in one of the two training contexts, which now contained one of the two previously encountered objects and a completely novel object (i.e., a wooden pyramid, 8 x 8 x 8 cm) at the same locations as the objects during training. We previously confirmed that mice do not display a preference for any of these objects (unpublished observations). Combinations of objects and contexts, the location of the novel object, the order of context exposures, and the test context were fully randomized across animals. Training and testing sessions were videotaped and object exploration times analyzed by a researcher blind to the experimental condition. To assess OR memory, a discrimination index (DI^OR^%) was calculated as the difference in time exploring the novel and familiar object, expressed as the ratio of the total time spent exploring both objects (i.e., (Time Novel - Time Familiar) / (Time Novel + Time Familiar) x 100%). A large DI^OR^% was interpreted as robust OR memory.

***Object re-exposure test***

Memory for the training objects *per se* after OiC training was also assessed with an object re-exposure test. Mice were handled (5 min per day for 5 days), habituated (3 days, 10 min each) and trained (10 min in each context) the same way as in the OiC task. For the object re-exposure test (14 days only), mice were re-exposed to one of the two training contexts with the identical object configuration. Training and testing sessions were recorded and object exploration times analyzed by a researcher blind to the experimental condition. Total object exploration time during the 10-min test session was compared with that of the training session to assess whether the mice remembered the objects seen during the training session, which would be evidenced by a reduced object exploration.

***Automated behavioral video analysis***

To standardize and confirm our manually scored behavioral findings with an automated analysis tool, DeepLabCut (version 2.2.0.4), an open source toolkit for markerless pose estimation, was used [1]. Nose, body center and the tail of the mice were labeled in more than 2,000 frames uniformly extracted from 157 different test videos, and 95% of the frames were used for training. A ResNet-50-based neural network [1] with 800,000 training iterations was applied to create new videos from these videos that contained frames in which the mice were close to the object. This allowed for further training of the network during object exploration epochs. From these new videos again more than 2,000 frames were extracted uniformly in DeepLabCut, in which the nose, body center and tail of the mice were labeled. The model was further trained with the additional labeled frames with 1,000,000 training iterations. A testing error of 1.56-2.06 pixels and a training error of 1.29-1.39 pixels was present in the final model. The p-cutoff for the X,Y-coordinates for subsequent analysis was set to higher than 0.95. To calculate total exploration times from the X and Y coordinates of the nose and body center, an in-house built pipeline in Python. Fiji (version 1.53t for Windows [2]) was used to select the objects and extract their pixel coordinates. Mice were considered to explore an object when their nose coordinates were within 2-3 pixels (corresponding to 1 cm) radius of the object with an estimation likelihood above the p-cutoff, while their body center coordinates were not in the area of the object itself (to exclude periods when the mouse was sitting on top of the object and sniffing air). Videos with apparent detection errors with the DeepLabCut analyses (e.g., due to the camera angle obstructing the detection of the nose and body center in the same frame) were excluded from the final analyses with the automated scoring, but not from manual scoring. We observed a significant correlation, of moderate strength, between manual and automated scoring for total object exploration time (*r*(60)= 0.74, *p* < 0.0001, Pearson correlation) as well as for the DI^OiC^% (*r*(95) = 0.69, *p* < 0.0001, Pearson correlation) [3]. Detailed inspection of the data points on which the manual and automated scorings deviated most, revealed that the manual scoring was less prone to errors, which is why the manual scorings were used for the primary analyses. The automated video analysis was used for comparing and verifying differences between experimental groups as well as for the analysis of exploration during training, and the measurement of total distance travelled during retention testing. Note that the video quality of experimental batches trained on weak OiC and tested at 3-day retention was insufficient for accurate DeepLabCut analysis, as indicated by significantly lower DeepLabCut estimation likelihood accuracy of nose detection compared to other experimental batches analyzed by DeepLabCut (U=951, *p*=0.006, two-tailed Mann-Whitney test), therefore was not included into the study. Of note, DeepLabCut analyses reproduced all our main observations on the effects of yohimbine administration after OiC training on 3-day and 14-day retention performance (Supplementary Figure 2A). Total distance travelled was calculated using X and Y coordinates of the nose identified by the DeepLabCut analysis of each video frame during the retention testing. Only frames in which the DeepLabCut estimation likelihood of accuracy was above 0.9 were included. The absolute differences between consecutive X and Y positions were summed across all frames to obtain the total distance travelled by each mouse.

***Immunohistochemistry***

All procedures were performed in the dark to avoid photobleaching. Sections were permeabilized with 0.1% Triton-X (Sigma-Aldrich) in PBS for 5 min, and blocked with 8% normal donkey serum (NDS, Sigma-Aldrich) and 0.3% Triton-X in PBS for 50 min. After washing in PBS (3 times, 5 min each), sections were incubated with primary antibody Guinea Pig anti-c-Fos (1:1,000, 226-308, Synaptic Systems) diluted in PBS with 2% NDS and 0.3% Triton-X overnight at 4 °C. The next day, sections were washed in PBS (3 times, 5 min each) and then incubated with fluorophore-conjugated secondary antibody Donkey anti-Guinea Pig Alexa 647 (1:750, 706-605-148, Jackson ImmunoResearch) in PBS with 2% NDS and 0.3% Triton-X for 2 h at room temperature. Lastly, sections were stained with 4′,6-diamidine-2′-phenylindole dihydrochloride (DAPI, 1:5,000, 62248, Thermo Scientific) for 1 min at room temperature and cover slipped with FluorSave reagent (Merck Millipore, #345789). Slides were stored at 4°C.

***Microscopy and Image analysis***

The overlap rate of the microscope was set as 20% to stich individual tile images. All tile images from one channel were merged to a stack, which was corrected for background and shading using the default settings of BaSiC plugin [4]. A column-by-column stitching was performed using the Grid/Collection Stitching plugin and channels were merged. When analyzing c-Fos immunostaining, a threshold was applied to the processed merged file and particles above the threshold, and larger than 20-pixel units were identified positive after a final confirmation of the cell based on DAPI staining. The threshold was kept constant across all animals within one staining batch, with all batches containing balanced group assignments. For tdTomato^+^ cells, all expressing cells were considered positive, regardless of labeling intensity. Images were analyzed by a researcher blind to the experimental condition. For prelimbic cortex (PL) images, a rectangular area of 250 x 150 μm^2^ was placed in layers 2/3 and a squared area of 250 x 250 μm^2^ was placed in layers 5/6 of the PL. For the distinction of the PL from the infralimbic area of the mPFC and the identification of PL sublayers, each analyzed mPFC image was compared and overlaid with the corresponding reference section of the interactive Allen Brain Atlas (<https://atlas.brain-map.org/>). For hippocampal images, the entire CA1, CA3, suprapyramidal and infrapyramidal blades of the dentate gyrus (DG) were defined as regions of interest (Supplementary Figure 3A). For all images, tdTomato^+^, c-Fos^+^ and tdTomato^+^+c-Fos^+^ cells were counted manually. For estimating the total number of DAPI^+^ cells in the entire dorsal hippocampus and PL, the number of DAPI^+^ cells was counted manually in an area of 100,000 μm^2^ in each subregion to calculate the local density of DAPI^+^ cells. This value was then multiplied by the area size of each subregion to estimate the total number of DAPI^+^ cells in the area.

As there are functional and anatomical differences within the subregions of the hippocampus and PL along the dorsoventral axis [5,6], we limited our analysis to the dorsal hippocampus and PL, given their well-established involvement in processing episodic(-like) information [7–9]. To minimize region-specific changes along the anteroposterior axis (AP) and allow for the averaging of our observations across slices within a single animal, the selection of sections used for immunohistochemistry was restricted to the dorsal hippocampus with the AP coordinates being between -1.70 to -2.30 mm from Bregma [7], and the PL with AP coordinates being +1.98 to +1.54 mm from Bregma [8,9]. Three sections were selected per animal, selected from one of the six series of brain slices that we cut. During image acquisition, we applied subsequent selection criteria, that is, we imaged brain sections that A) spanned the AP-axis of interest and B) contained all subregions/sublayers of the dorsal hippocampus and PL. Images that did not meet the pre-defined criteria were excluded from further analysis, resulting in the analysis of at least 2 images per brain region per animal. As mixed-effects analysis in the pilot data set, characterizing tdTomato labeling in home-cage versus trained mice (Supplementary Figure 3D), indicated no main effect of either laterality (dorsal hippocampus: *F*_1,14_=1.86, *p*=0.19, prelimbic cortex: *F*_1,14_=0.68, *p*=0.42) or slice position along the AP-axis (dorsal hippocampus: *F*_2,28_=2.19, *p*=0.13, prelimbic cortex: *F*_2,28_=1.007, *p*=0.38), for all subsequent experiments we imaged the dorsal hippocampus and prelimbic cortex unilaterally. Note that a two-way RM analysis of images obtained from a random subgroup of mice (n=10) from each experimental group demonstrated no main effect of AP position for any of our readouts (%Fos^+^, %tdTomato^+^ cells and the reactivation rate) in either in the dorsal hippocampus (%Fos^+^: *t*_9_=0.48, *p*=0.64; %tdTomato^+^: *t*_9_=0.72, *p*=0.49; RR: *t*_9_=0.28, *p*=0.79) or prelimbic cortex (%Fos^+^: *t*_9_=0.56, *p*=0.59; %tdTomato^+^: *t*_9_=1.54, *p*=0.16; RR: *t*_9_=1.73, *p*=0.12). These findings further indicate that the measurements of readouts were consistent among the analyzed AP ranges.

The reactivation rate (RR) was defined as the percentage of tdTomato^+^ cells that was also c-Fos^+^ [RR = (number of tdTomato^+^+c-Fos^+^ cells) / (number of tdTomato^+^ cells) * 100%] as used before[10,11]. The percentage of observed overlap between tdTomato and c-Fos signals [overlap = (number of tdTomato^+^+c-Fos^+^ cells) / (number of DAPI^+^ cells) * 100%] was compared to the expected overlap based on chance [overlap by chance = (number of c-Fos^+^ cells / number of DAPI^+^ cells) * (number of tdTomato^+^ cells / number of DAPI^+^ cells) * 100%].

***Statistical analyses***

Data was first subjected to a normality test prior to further comparisons (Shapiro-Wilk normality test; alpha = 0.05).

If the data followed normal distribution;

After performing outlier analyses (Grubbs’ (alpha = 0.05), or ROUT (Q = 1%) method), one-way ANOVAs were used to compare more than two experimental groups in the OiC task (saline, 0.3 mg/kg or 1 mg/kg yohimbine as a fixed factor), which was followed by a *post-hoc* Tukey’s HSD test. Cell counts were compared by using a two-way ANOVA (testing delay (recent, remote) x yohimbine treatment (saline, 0.3 mg/kg yohimbine)) followed by *post-hoc* Sidac tests or mixed-effects RM analysis tests (testing delay (recent, remote) x brain region (dorsal hippocampus, PL)) followed by independent samples *t-*tests. Two-tailed paired *t*-tests were used to compare total object exploration times during training vs testing on the object re-exposure test, as well as to compare chance overlap versus observed overlap rates in histology data. One-sample *t*-tests were used to compare the DI%s to a hypothetical value (i.e., 0) in the OiC task and OR test. An independent samples *t*-test was used to compare the DI^OiC^% at the 14-day vs 10-day retention tests.

If the data deviated from a normal distribution;

Non-parametric tests, i.e., Kruskal Wallis test for comparing more than two experimental groups (alternative to one-way ANOVA test), Wilcoxon matched pairs signed-rank test for paired comparisons (alternative to two-tailed paired *t*-tests) and Wilcoxon signed rank test for comparisons to a hypothetical value (alternative to one-sample *t*-tests) were applied.

Pearson correlation tests were used to compute correlations between the variables. *P*-values < 0.05 were considered significant. Correlations were statistically compared according to Lenhard and Lenhard (2014) [12].

**Supplementary Figures**

**
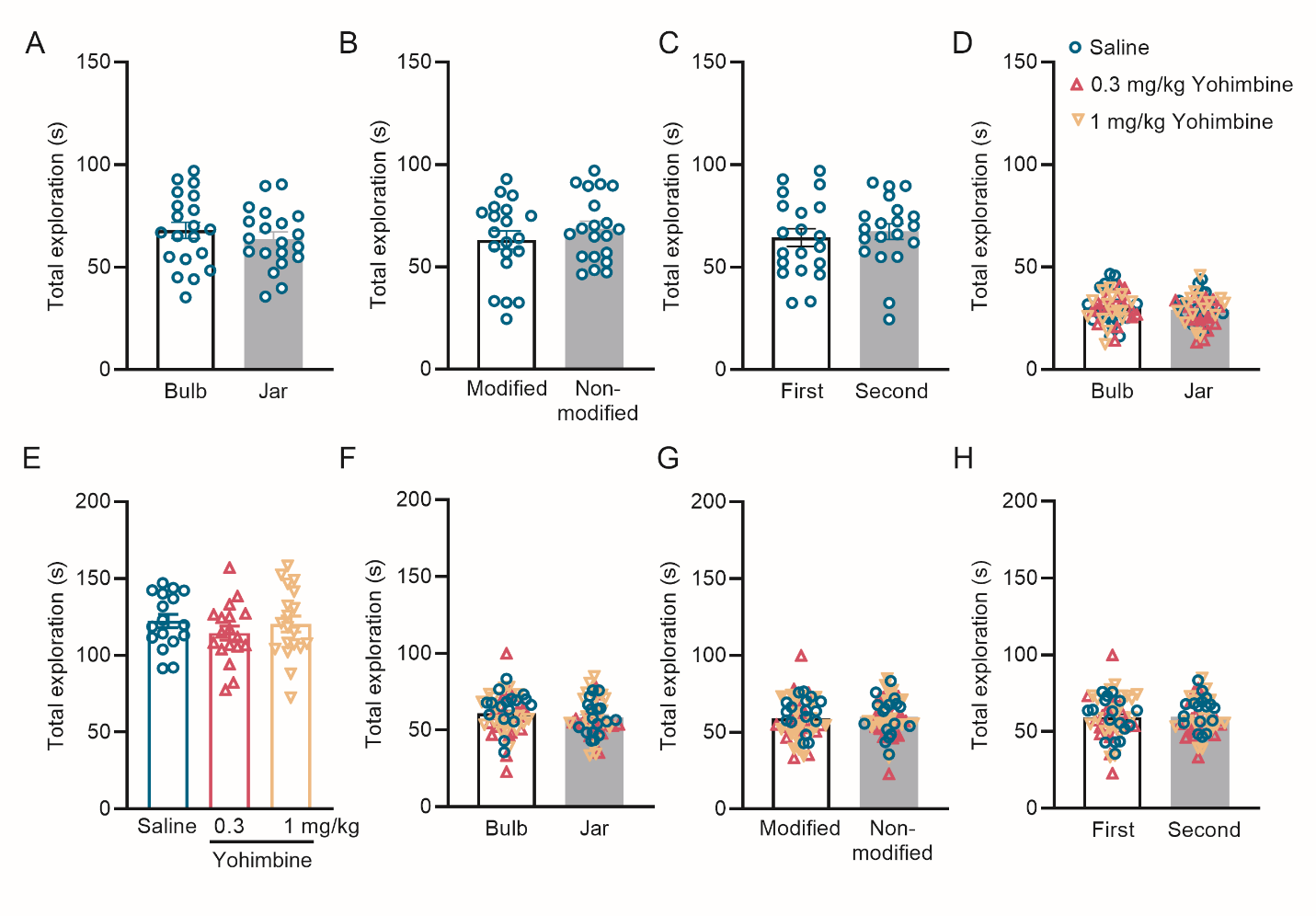
**

**Supplementary Figure S1.** Control measures for object-in-context (OiC) or object re-exposure trainings. (A) Total exploration duration of each object during OiC training (*t*_24_=1.50, *p*=0.15, paired *t*-test) in saline-treated mice used for 14-day retention testing in Figure 1. (B) Total exploration duration of objects in either contextually modified or non-modified context during OiC training (*t*_10_=1.55, *p*=0.13, independent samples *t*-test) in saline-treated mice used for 14-day retention testing in Figure 1. (C) Total exploration duration of objects in either contexts visited as the first vs second order during OiC training order (*t*_15_=0.13, *p*=0.90, paired *t*-test) in saline-treated mice used for 14-day retention testing in Figure 1. (D) Total exploration duration of each object during object re-exposure test (*t*_52_=0.48, *p*=0.64, paired *t*-test) in all saline- and yohimbine (0.3 mg/kg and 1 mg/kg) mice. (E) Total exploration duration of objects of mice administered with yohimbine (0.3 mg/kg or 1 mg/kg) or saline during OiC training (*F*_2,51_=0.73, *p*=0.49, one-way ANOVA). (F) Total exploration duration of each object in yohimbine (or saline)-treated groups during OiC training (*t*_53_=1.25, *p*=0.22, paired *t*-test). (G) Total exploration duration of objects in either contextually modified or non-modified context in yohimbine (or saline)-treated groups during OiC training (*t*_33_=0.21, *p*=0.83, unpaired *t*-test). (H) Total exploration duration of objects in either contexts visited as the first vs second order during OiC training of yohimbine (or saline)-treated groups (*t*_53_=0.17, *p*=0.86, paired *t*-test). Data represent means ± standard error of the mean and all data points.


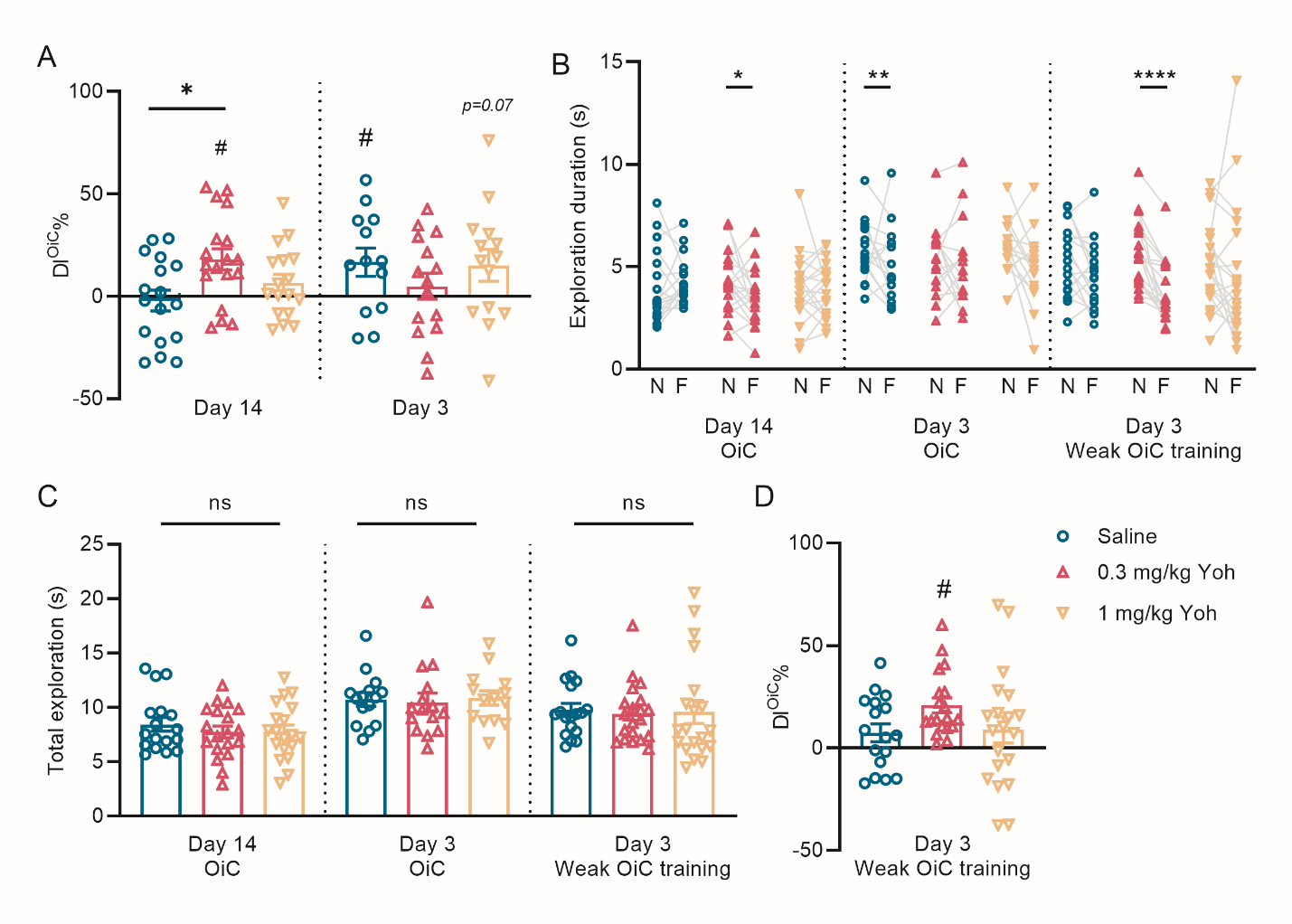


**Supplementary Figure S2.** Verification of yohimbine effects identified in this study by the automated video analysis tool DeepLabCut and control measures for yohimbine effects on OiC memory. (A) Discrimination indexes (DI^OiC^%) of yohimbine (0.3 mg/kg or 1 mg/kg) or saline-treated mice at the 14-day or 3-day retention testa analyzed by DeepLabCut (14-day: main effect of yohimbine administration *F*_2,49_=4.41, *p*=0.02, one-way ANOVA, saline vs 0.3 mg/kg yohimbine-treated: *p*=0.01; 3-day: main effect of yohimbine administration *F*_2,45_=0.147, *p*=0.87, one-way ANOVA). (B) Total exploration times of the object that was novel (N) and familiar (F) in the test context during the retention tests. At the 14-day retention test, mice treated with saline or 1 mg/kg yohimbine spent similar time exploring the two objects (saline: *t*_16_=0.63, *p*=0.53, 1 mg/kg yohimbine: *t*_18_=0.20, *p*=0.85, paired t-test), whereas mice treated with 0.3 mg/kg yohimbine spent significantly more time exploring the object novel to the text context (*t*_18_=2.13, *p*=0.047), consistent with the findings of DI^OiC^ analysis (Figure 2B). At the 3-day retention test, saline-treated mice showed preferential exploration of the novel relative to the familiar object in the test context (*t*_14_=3.08, *p*=0.008), whereas yohimbine-treated groups spent similar time exploring the two objects (0.3 mg/kg: *t*_14_=0.20, *p*=0.85, 1 mg/kg: *t*_14_=2.06, p=0.06), also consistent with the findings of DI^OiC^ analysis (Figure 2B). During the 3-day retention test of weak OiC training, mice administered 0.3 mg/kg yohimbine spent significantly more time exploring the novel object in that context (*t*_18_=5.71, *p*<0.0001), whereas saline- or 1 mg/kg yohimbine-treated groups spent similar time exploring the two objects (saline: *t*_16_=1.83, *p*=0.09, 1 mg/kg yohimbine: *t*_19_=0.68, *p*=0.51), also consistent with the findings of DI^OiC^% analysis (Figure 2B). (C) Total exploration of both objects during retention test in saline and yohimbine-treated mice (Day 14 OiC: *H*=0.25, *p*=0.88; Day 3 OiC: *H*=1.07, *p*=0.58; Day 3 Weak OiC: *H*=1.46, *p*=0.48, Kruskal-Wallis test). (D) Discrimination indexes (DI^OiC^%) of saline or yohimbine (0.3 mg/kg or 1 mg/kg) treated mice at the 3-day retention test after a weak OiC training protocol (main effect of yohimbine administration *F*_2,53_=2.13, *p*=0.19, one-way ANOVA). Data represent means ± standard error of the mean and all data points.


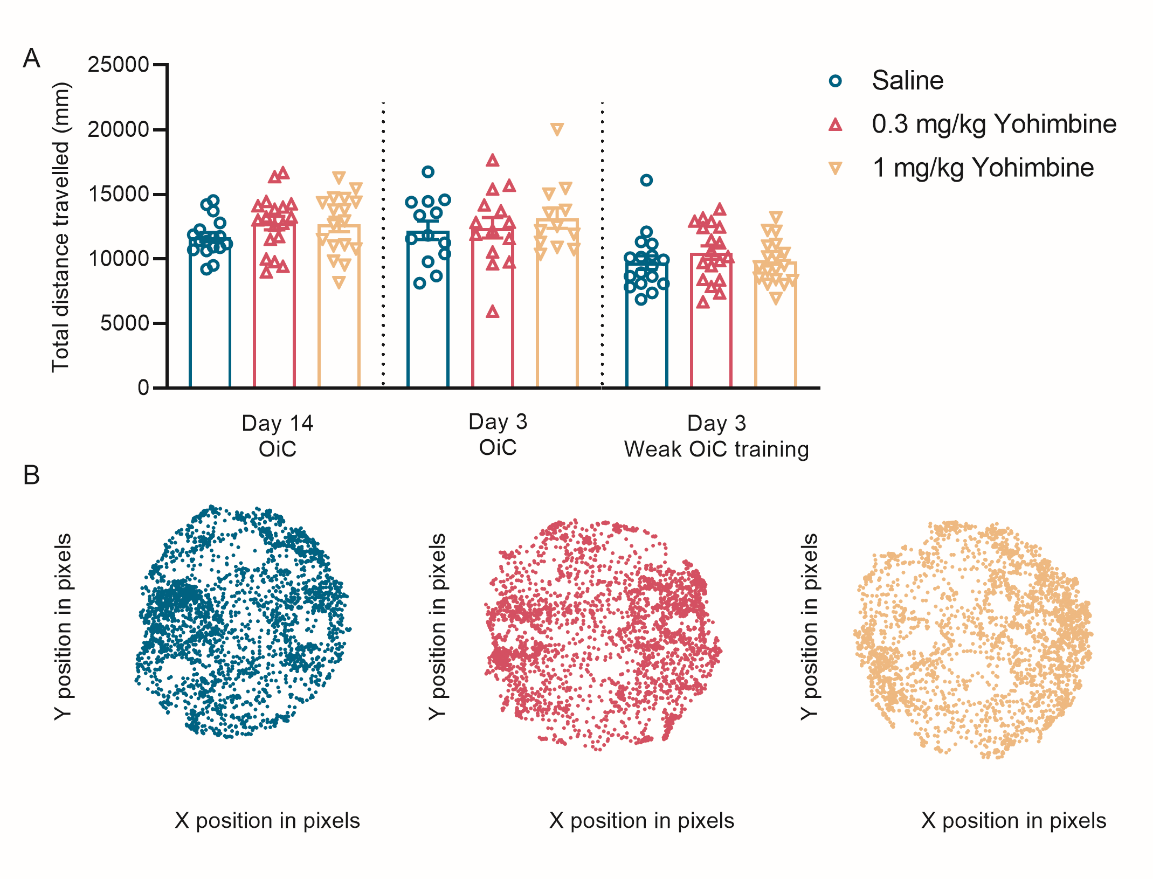


**Supplementary Figure S3.** Total distance travelled during the object-in-context (OiC) retention tests.. (A) Total distance travelled by saline- and yohimbine-treated mice (0.3 mg/kg or 1 mg/kg) during the retention tests (14-day OiC: *F*_2,49_=1.48, *p*=0.24; 3-day OiC: *F*_2,36_=0.41, *p*=0.67; 3-day weak OiC: *F*_2,50_=0.80, *p*=0.46, one-way ANOVA). (B) Representative travel trajectories of saline- and yohimbine-treated mice (0.3 mg/kg or 1 mg/kg) during the 3-day retention test. Data represent means ± standard error of the mean and all data points.


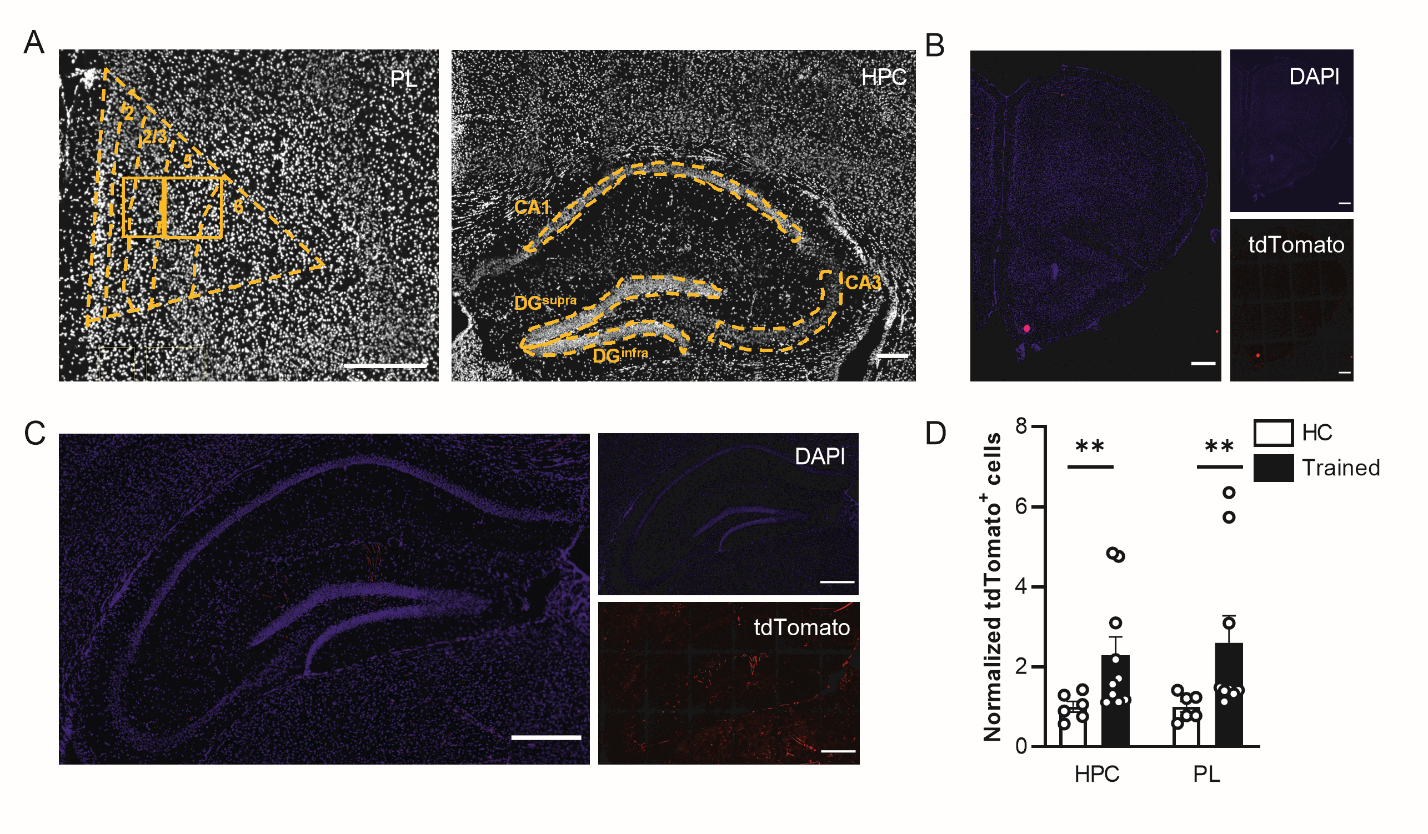


**Supplementary Figure S4.** Characterization of tdTomato labeling in the prelimbic cortex (PL) and dorsal hippocampus (HPC). (A) Schematic representation of the region of interests (highlighted by yellow dashed lines) in the PL and dorsal hippocampus analyzed in this study. Scale bars represent 50 µm. (B) Representative images showing tdTomato^+^ cells at 72 h posttraining in the PL without 4-hydroxytamoxifen (4-OHT) administration, serving as negative controls. Given that the tdTomato expression depends on 4-OHT administration, tdTomato expression without the 4-OHT is negligible. Scale bars represent 50 µm. (C) Representative images showing tdTomato^+^ cells at 72 h posttraining in the dorsal hippocampus without 4-OHT administration, serving as negative controls. Given that the tdTomato expression depends on 4-OHT administration, tdTomato expression without the 4-OHT is negligible. Scale bars represent 50 µm. (D) Number of tdTomato^+^ neurons in 100,000 μm^2^ normalized to the home-cage (HC) conditions in the dorsal hippocampus and PL in HC vs trained conditions (*F*_1,14_=5.45, *p*=0.03, mixed-effects RM analysis (training); followed by Mann-Whitney test PL: ***p*=0.007, HPC: ***p*=0.001). Overall numbers of tdTomato^+^ cells in dorsal hippocampal subregions both in HC and OiC-trained conditions were relatively low with on average 0.1%, 0.3%, and 0.3% of cells being labeled in the CA1, CA3 and DG respectively (data not shown), indicating that a rather small proportion of all activated hippocampal cells (expected to be 16%, 10% and 2% respectively [13]) were labelled by tdTomato. Therefore, we analyzed tdTomato^+^ cells in the total dorsal hippocampus without further distinction of the subregional contributions. Data represent means ± standard error of the mean and all data points.

**
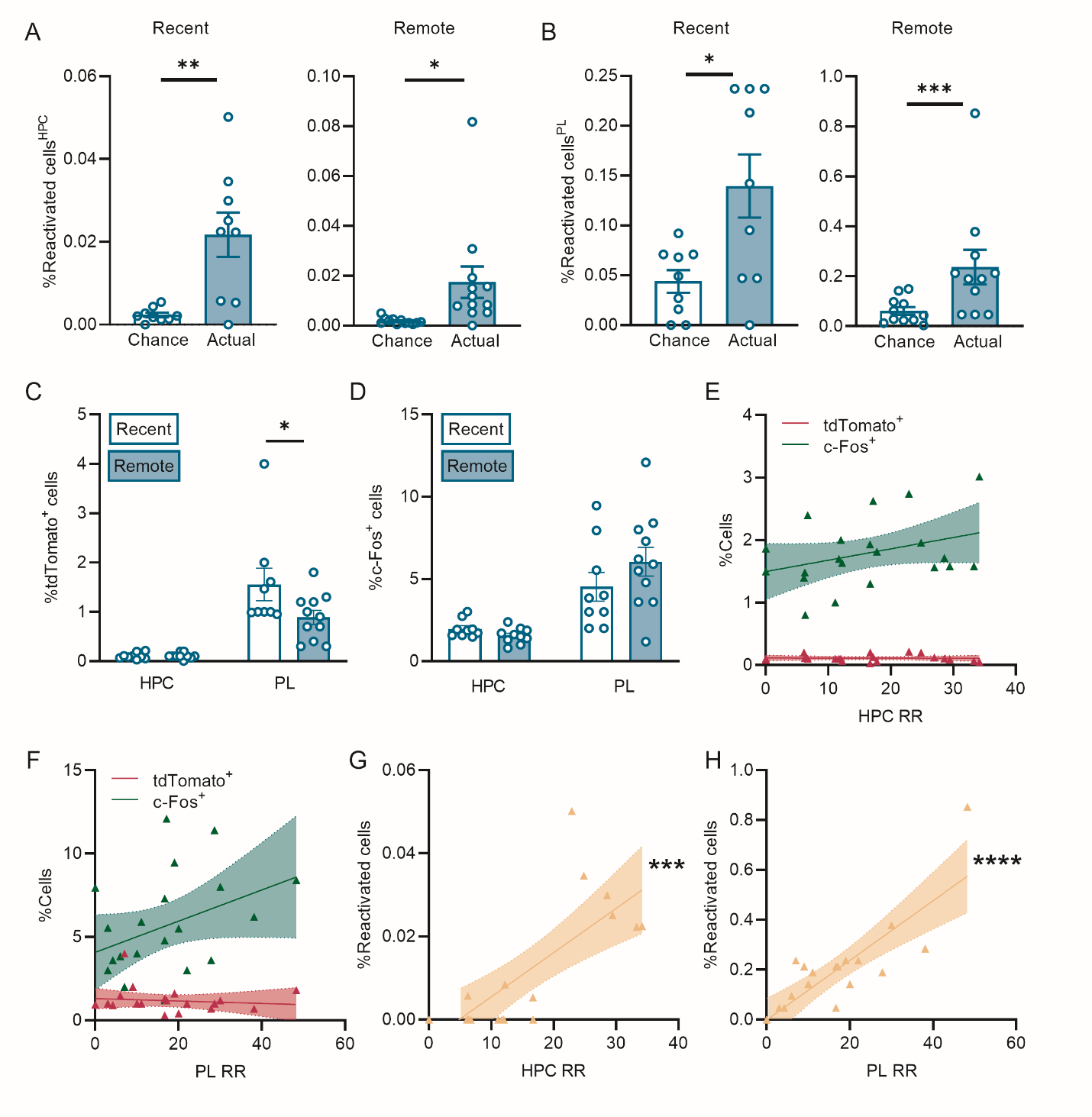
Supplementary Figure S5.** Additional measures for reactivation rate (RR) assessment in the dorsal hippocampus (HPC) and prelimbic cortex (PL) at recent (3-day) and remote (14-day) retention tests in saline-treated mice. (A) Percentage of reactivated cells that would occur by chance vs the actually observed rate in the dorsal hippocampus during the 3-day and 14-day OiC retention test (3-day: *t*_8_=3.83, ***p*=0.005, paired *t*-test, 14-day: *t*_11_=2.63, **p*=0.02, paired *t*-test). (B) Percentage of reactivated cells that would occur by chance vs the actually observed rate in the PL during 3-day and 14-day OiC retention test (3-day: *t*_8_=2.83, **p*=0.02, paired *t*-test, 14-day: *W*=66.0, ****p*=0.001, Wilcoxon matched-pairs signed rank test). (C) Percentage of tdTomato^+^ cells in the dorsal hippocampus and PL at 3-day vs 14-day OiC retention test (*F*_1,16_=7.38, *p*=0.02 (testing delay*brain region), *F*_1,16_=49.74, *p*<0.0001 (brain region), *F*_1,21_=6.89, *p*=0.02 (testing delay), mixed effects analysis followed by multiple comparisons: HPC: *p*=0.99, PL: *^**^p*=0.001). (D) Percentage of c-Fos^+^ cells in the dorsal hippocampus and PL at 3-day vs 14-day retention test (*F*_1,37_=1.21, *p*=0.28 (testing delay*brain region), *F*_1,37_=27.03, *p*<0.0001 (brain region), *F*_1,37_=27.03, *p*=0.43 (testing delay), mixed effects analysis). (E) Correlational analysis between the reactivation rate and percentage of c-Fos^+^ or tdTomato^+^ cells in the dorsal hippocampus (c-Fos: *r*_21_=0.35, *p*=0.12; tdTomato: *r*_21_=-0.03, *p*=0.89, Pearson correlations). (F) Correlational analysis between the reactivation rate and percentage of c-Fos^+^ or tdTomato^+^ cells in the PL (c-Fos: *r*_21_=0.38, *p*=0.09; tdTomato: *r*_21_=-0.12, *p*=0.62, Pearson correlation). (G) Correlational analysis between the reactivation rate and percentage of reactivated cells (tdTomato^+^+c-Fos^+^) in the dorsal hippocampus (*r*_21_=0.74, ****p*=0.0001, Pearson correlation). (H) Correlational analysis between the reactivation rate and percentage of reactivated cells (tdTomato^+^+c-Fos^+^) in the PL (*r*_21_=0.81, *****p*<0.0001, Pearson correlation). Data represent means ± standard error of the mean and all data points.


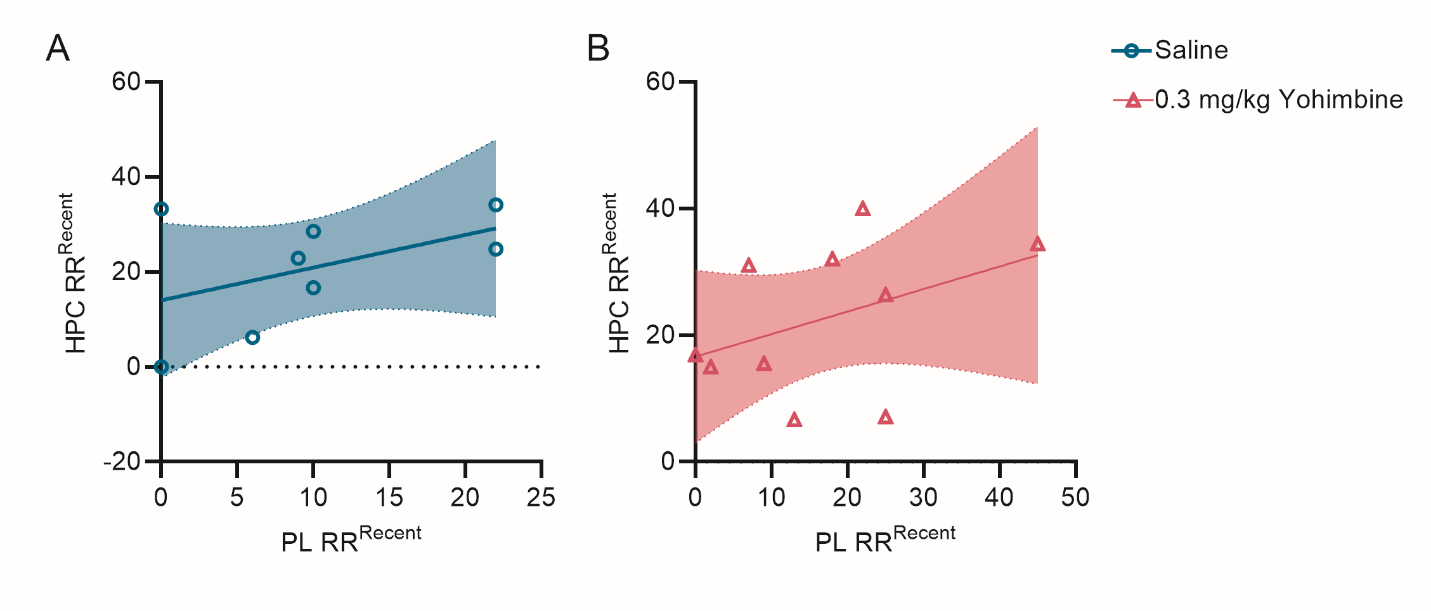


**Supplementary Figure S6.** Additional analysis of the activated cell populations in the dorsal hippocampus (HPC) and prelimbic cortex (PL) over time. (A) Correlational analysis between the reactivation rates (RR) of engram cells located in the dorsal hippocampus vs PL of saline-treated mice at 3-day OiC retention test (*r*_6_=0.48, *p*=0.24, correlation analysis). (B) Correlational analysis of the RRs of engram cells located in the hippocampus vs PL of 0.3 mg/kg yohimbine-treated mice at 3-day OiC retention test (*r*_8_=0.41, *p*=0.24, Pearson correlation). Data represent means ± standard error of the mean and all data points.

**References**

1. Mathis A, Mamidanna P, Cury KM, Abe T, Murthy VN, Mathis MW, et al. DeepLabCut: markerless pose estimation of user-defined body parts with deep learning. Nat Neurosci. 2018;21.

2. Schindelin J, Arganda-Carreras I, Frise E, Kaynig V, Longair M, Pietzsch T, et al. Fiji: An open-source platform for biological-image analysis. Nat Methods. 2012.

3. Akoglu H. User’s guide to correlation coefficients. Turk J Emerg Med. 2018;18.

4. Peng T, Thorn K, Schroeder T, Wang L, Theis FJ, Marr C, et al. A BaSiC tool for background and shading correction of optical microscopy images. Nature Communications 2017 8:1. 2017;8:1–7.

5. Strange BA, Witter MP, Lein ES, Moser EI. Functional organization of the hippocampal longitudinal axis. Nature Reviews Neuroscience 2014 15:10. 2014;15:655–669.

6. Heidbreder CA, Groenewegen HJ. The medial prefrontal cortex in the rat: evidence for a dorso-ventral distinction based upon functional and anatomical characteristics. Neurosci Biobehav Rev. 2003;27:555–579.

7. Fanselow MS, Dong HW. Are the dorsal and ventral hippocampus functionally distinct structures? Neuron. 2010;65:7–19.

8. Matos MR, Visser E, Kramvis I, van der Loo RJ, Gebuis T, Zalm R, et al. Memory strength gates the involvement of a CREB-dependent cortical fear engram in remote memory. Nature Communications 2019 10:1. 2019;10:1–11.

9. Lee JH, Kim W Bin, Park EH, Cho JH. Neocortical synaptic engrams for remote contextual memories. Nat Neurosci. 2023;26.

10. Gulmez Karaca K, Brito DVC, Kupke J, Zeuch B, Oliveira AMM. Engram reactivation during memory retrieval predicts long-term memory performance in aged mice. Neurobiol Aging. 2021. 2021. https://doi.org/10.1016/j.neurobiolaging.2021.01.019.

11. Gulmez Karaca K, Kupke J, Brito DVC, Zeuch B, Thome C, Weichenhan D, et al. Neuronal ensemble-specific DNA methylation strengthens engram stability. Nat Commun. 2020;11.

12. Lenhard W, Lenhard A. Hypothesis Tests for Comparing Correlations. . Psychometrica. 2014. 2014.

13. Garner AR, Rowland DC, Hwang SY, Baumgaertel K, Roth BL, Kentros C, et al. Generation of a synthetic memory trace. Science (1979). 2012;335:1513–1516.
